# Sex-dimorphism in human serum endocannabinoid and n-acyl ethanolamine concentrations across the lifespan

**DOI:** 10.1038/s41598-023-50426-4

**Published:** 2023-12-27

**Authors:** Khalisa Amir Hamzah, Leisa-Maree Toms, Nathaniel Kucharski, Julia Orr, Natalie P. Turner, Peter Hobson, David S. Nichols, Luke J. Ney

**Affiliations:** 1 School of Psychology and Counselling, Faculty of Health, Queensland University of Technology, 149 Victoria Park Road, Kelvin Grove, Brisbane, 4059 Australia; 2 School of Public Health and Social Work, Faculty of Health, Queensland University of Technology, Kelvin Grove, Australia; 3 The University of Queensland, Queensland Alliance for Environmental Health Sciences, Woolloongabba, QLD Australia; 4 The Centre for Children’s Health Research (CCHR), Queensland University of Technology, 62 Graham Street, South Brisbane, QLD 4101 Australia; 5 Sullivan and Nicolaides Pathology, 24 Hurworth Street, Bowen Hills, QLD 4006 Australia; 6 Central Science Laboratory, University of Tasmania, Sydney, Australia

**Keywords:** Biochemistry, Endocrinology

## Abstract

The endocannabinoid (ECB) system has recently been considered a potential treatment target for various clinical disorders. However, research around age- and sex-related changes within the ECB system is relatively limited. To improve our understanding of these changes, the current study measured arachidonoyl ethanolamide (AEA), 2-arachidonoyl glycerol (2-AG), oleoylethanolamine (OEA), palmitoylethanolamine (PEA), arachidonic acid (AA), cortisol, and progesterone in pooled serum samples stratified by sex (male and female) and age groups (5–15; 15–30; 30–45; 45–60; 60–75; 85+), using liquid-chromatography tandem mass spectrometry. Serum progesterone levels significantly increased in females of the 15–30 and 30–45 age groups, before declining. Significantly higher cortisol, AEA, 2-AG, OEA, and PEA were found in males and in older age, while significantly higher AA was found in females. Our results indicate that ECBs and related hormones exhibit sexual dimorphism in the age ranges that correspond with female pregnancy, menopause, and post menopause. Male testosterone levels most likely influences male ECB changes throughout the lifespan. Future research could capitalise on these findings by performing repeated measurements in individuals in a longitudinal style, to further refine the temporal profile of age-specific changes to the ECB system identified here.

## Introduction

The endocannabinoid (ECB) system is an important lipid signalling system, of which arachidonoylethanolamide (AEA) and 2-arachidonylglycerol (2-AG) are the primary compounds^1,2^. These ECBs are produced in postsynaptic neurons and released into the presynaptic space, travelling backwards towards the presynaptic terminal^3,4^. There, they interact with their presynaptic receptors, which can be broadly construed as CB_1_ for the central nervous system and digestive organs, and CB_2_ for the regulation of immunity and inflammation^2,5^. ECBs are produced in response to various stimuli and are rapidly degraded by enzymes to prevent overstimulation of the ECB system. Notably, both AEA and 2-AG are degraded via hydrolysis to arachidonic acid (AA), which is also a precursor for 2-AG synthesis. ECBs play a crucial role in the homeostasis of the body^1^. For example, hyperactivity of ECB signalling (i.e., an increased production of ECBs) influences excessive intake and storage of high-calorie foods, while hypoactivity (decreased production) may contribute to the risk of developing major depressive, anxiety, and post-traumatic stress disorders^6^. The ECB system has also recently been implicated in various affective and cognitive processes including mood, appetite, and pain sensation, and the development and symptomatology of psychotic disorders^7–10^. As such, ECBs are considered to be potential treatment targets for various clinical disorders.

Although research is limited, the ECB system appears to exhibit a temporal profile that fluctuates over the lifetime. It is very likely that this affects the delicate balance of production, signalling, and regulation of ECBs. When examined in the brains of middle-aged mice, it was found that 2-AG levels decreased significantly in the hippocampus and moderately in the caudate putamen, along with AEA levels that decreased in the medial prefrontal lobe and cingulate cortex slightly earlier in age^11^. This suggests an association of local 2-AG and AEA levels with cognitive function. Additionally, hippocampal 2-AG levels decreased with age in older mice, which mirrored an age-related decrease in diacylglycerol lipase α (DAGLα), the major synthesising enzyme for 2-AG^12^. In contrast, monoacylglycerol lipase (MAGL), the major degrading enzyme for 2-AG, increased as the mice got older^12^. Also, as age increased, it was found that levels of the CB_1_ receptors decreased prominently in the cerebellum, cerebral cortex, hippocampus, limbic and hypothalamic structures of rodents^13^. ECBs were also reported to be less likely to bind to CB_1_ receptors in older age^14^, suggesting that, regardless of ECB circulating levels, as age increases, CB receptors are less sensitive to ECB signalling. However, to our knowledge there have been very few reports of age-related effects on ECB expression in humans. In one study, Fanelli et al.^15^ reported that 2-AG and palmitoylethanolamine (PEA) were higher in older human females, though no differences were observed in circulating ECB levels across different ages in males.

Similar to this finding, there is substantial emerging evidence that the ECB system is sexually dimorphic^16^; however, there is conflicting evidence as to in which direction. Blanton et al.^17^ reported that female rodents tended to have higher circulating levels of AEA compared to males, and female rats have also been found to have higher levels of 2-AG in the hypothalamus^18^ and AEA and 2-AG in the pituitary^19^, relative to male rats. On the contrary, analysis of human plasma samples has found no differences observed between males and females for AEA, oleoylethanolamine (OEA), and PEA, though males presented significantly higher 2-AG and 1-AG levels than females^15^. In a meta-analysis, AA was reported to be significantly higher in females compared to males^20^. These differences may be primarily due to two reasons: the circulating levels of ECBs in plasma samples comprises of a different concentration of ECBs than of those in brain tissue^21^; and while animal research can greatly inform clinical human research, the translatability of ECB concentrations may be limited^17,22^.

Evidence supporting the sex differences of ECBs in humans is still extremely limited, though there is some emerging evidence that ECB levels are higher in saliva and lower in hair samples of males compared to females^23–26^. While rodents are some of the most utilised model organisms for clinical research, a clear translation and extrapolation of findings to humans is not always possible, and blood concentrations of ECBs between the sexes has been rarely studied. As such, future studies of both sex and age effects on ECBs have a distinct call for investigation. In the current study, we measured AEA, 2-AG, OEA, PEA, AA, cortisol, and progesterone in pooled serum samples (total N = 700) that were stratified by sex and collected cross-sectionally across the lifespan (ages 5 until over 85). We hypothesised that 2-AG and AA (but not the ethanolamines) would be higher in males compared to females, and that ECB concentrations would decrease with increasing age.

## Method

### Participant samples

The use of human blood samples in this study was approved by the Human Research Ethics Committee (approval number 1086), and all methods were performed in accordance with these relevant guidelines and regulations. Informed consent was obtained from all subjects and/or their legal guardians. Samples for the current study were collected using the methods described in Toms et al.^27^. This method involves collaboration with one of the key national pathology laboratories in Australia (Sullivan Nicolaides Pathology, Australia), who have archived pooled and de-identified human blood sera in an ongoing series of human biomonitoring studies from 2002 until the present^28,29^.

In the current study, 7 age ranges were stratified by male and female sex (Table 1), with two pooled clusters of 25 participants in each stratified bracket. This resulted in 50 participants per sex-stratified age range, 28 variable groups in total, and a total of 700 Australian participants across an age range of 5 years to over 85 years of age. 2 mL of pooled serum were available in each stratified sample and were aliquoted from a larger sample base at Sullivan Nicolaides Pathology, Brisbane. Specific demographics of participants are not provided in this collection method, therefore information such as participant body-mass index, ethnicity, medicine use, and so on were not available.

**Table 1:** Average age of participants across sex and age range stratifications.

	Males	Females	Total
5–15	11.94	11.51	11.72
15–30	21.38	23.76	22.57
30–45	37.34	35.96	36.65
45–60	52.71	52.57	52.64
60–75	67.89	67.28	67.58
75–85	79.29	79.91	79.60
85+	88.66	88.29	88.48

Standard deviations are not reported because only average age values for each pool were recorded. Each cell in Male and Female columns contains serum samples from two pooled clusters of 25 participants, equalling 50 participant samples per cell in the table.

### Sample collection and storage

All samples were collected in 2021 to 2022. Blood sera were collected from routine blood collections at multiple pathology laboratories in Australia (Sullivan Nicolaides Pathology, Australia). Samples were collected in serum separating tubes, centrifuged, and stored at − 20 °C immediately. Sera were thawed for sample pooling, and refrozen at − 20 °C.

### Chemical analysis

Serum samples were prepared and analysed using liquid chromatography tandem mass spectrometry (LC–MS/MS) as previously described^30,31^. Briefly, 1.6 mL of 50:50 ethyl acetate: cyclohexane extraction solution and 20 µL of labelled isotopic standards (d4-AEA, d5-2AG, d4-OEA, d4-PEA, d8-AA, d4-cortisol, and d9-progesterone) were added to 400 µL of serum from each pooled sample. The resulting solution was vortexed and centrifuged, after which the organic supernatant layer was transferred to a HPLC vial, evaporated using a vacuum evaporator and reconstituted in a final solution of 15 µL acetonitrile. 5 µL of this solution was injected to a Nexera X2 UHPLC comprising of two binary pumps and a column oven, coupled with a SCIEX Triple Quadrupole Trap 6500 mass spectrometer. Mobile phase A consisted of 2 mM ammonium acetate and mobile phase B was 100% acetonitrile, with a total runtime of 12 min. Our method is linear and has lower limits of quantification suitable for the current application, previously reported by Ney et al.^30^. Notably, a change of instrumentation in the current study has resulted in higher sensitivity and lower limits of detection compared to what we have previously reported.

The limits of quantification (LOQ) using this new instrument were estimated by calculating the variability in peak area intensity of the isotopically labelled standards that were spiked into the samples in this study. The LOQ was estimated as two times the limit of detection, which was determined as three times the standard deviation from the peak area intensity of each isotopically labelled standard across the samples in the study, with the exceptions of AEA and OEA, which were spiked too high to gain an accurate estimate of LOQ in the current study. In these cases, the LOQs from our establish method are cited, see Ney et al.^30^. The LOQ from this experiment were therefore 482.41 pg/mL for AA, 277.91 pg/mL for 2-AG, 24 pg/mL for AEA, 30 pg/mL for OEA, 135 pg/mL for PEA, 46 pg/mL for progesterone, and 856.54 pg/mL for cortisol.

### Statistical analysis

To test the effects of age and sex on serum levels of AEA, 2AG, OEA, PEA, AA, cortisol, and progesterone, 7(Age: 5–15 years, 15–30 years, 30–45 years, 45–60 years, 60–75 years, 75–85 years, 85 + years) × 2(Sex: Male, Female) univariate ANOVAs were conducted for each analyte separately. Partial eta-squared (η_p_^2^) effect sizes were calculated and reported, with the alpha level of the tests set to 0.05. Bivariate correlations (Pearson’s coefficient correlations) were conducted to examine the relationships between the measured biomarkers, with correlations across the entire sample as well as within Male and Female sexes performed separately. It should be noted that, due to the use of pooled data, all statistical analyses are underpowered and have inflated effect sizes^32^, despite the actual sample sizes being adequate and most likely reflective of the general population. All analyses were conducted using SPSS v29 for Windows. Quantitative LC–MS/MS data was imported into RStudio (v2023.06.0 Build 421) and the package mixOmics (https://github.com/mixOmicsTeam/mixOmics) was used to generate unsupervised principal component analysis (PCA) plots grouped by sex and age.

## Results

### Hormones

**Figure 1:**
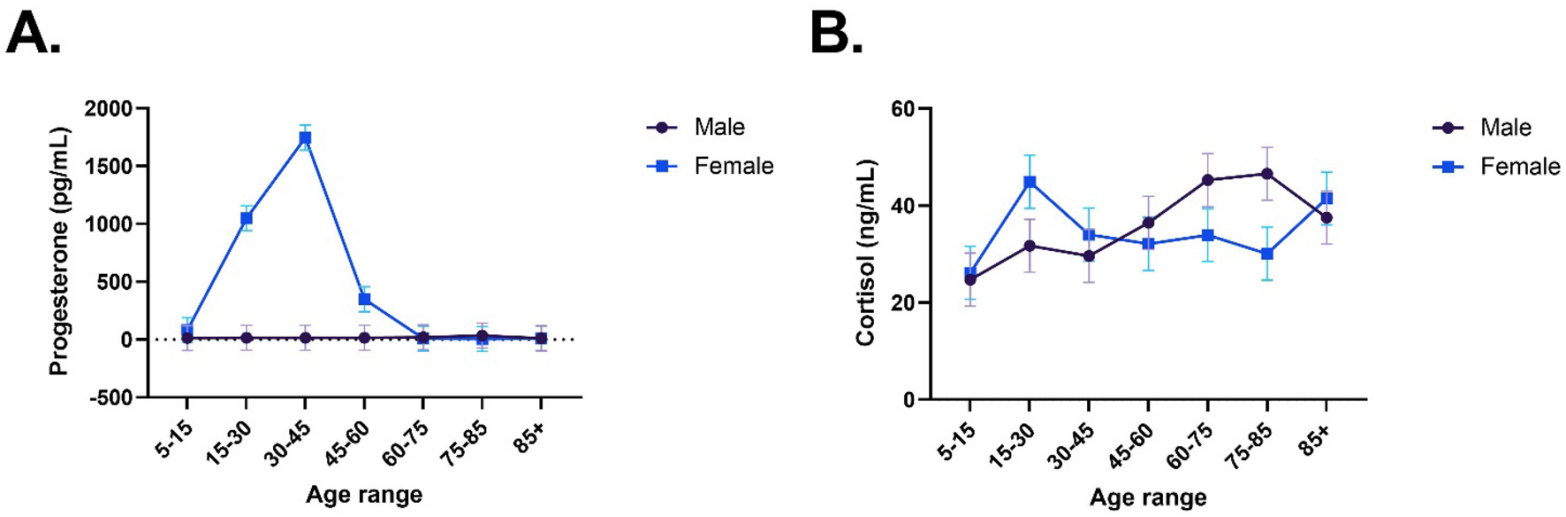
Average levels of serum progesterone (**A**) and cortisol (**B**) levels across the lifespan, stratified by sex. Note: each data point is pooled serum from 50 participants. Error bars are standard error.

Separate univariate ANOVAs were conducted for each dependent variable. As expected, significant increases in serum progesterone levels were observed in females in the 15–30 and 30–45 age groups, before declining in the later ages (Fig. 1A). This was reflected by significant Sex: *F*(1,14) = 59.90, *p* < 0.001, *η*_*p*_^*2*^ = 0.81, Age: *F*(6,14) = 19.73, *p* < 0.001, *η*_*p*_^*2*^ = 0.89, and Sex × Age: *F*(6,14) = 19.93, *p* < 0.001, *η*_*p*_^*2*^ = 0.90 effects. This analysis was a confirmation that our analysis of the serum samples was successful. No significant effects were found for cortisol levels (Fig. 1B), with Sex: *F*(1,14) = 0.21, *p* = 0.658, *η*_*p*_^*2*^ = 0.01, Age: *F*(6,14) = 1.84, *p* = 0.173, *η*_*p*_^*2*^ = 0.44, and Sex × Age: *F*(6,14) = 1.72, *p* = 0.188, *η*_*p*_^*2*^ = 0.43 all non-significant.

### ECBs

**Figure 2:**
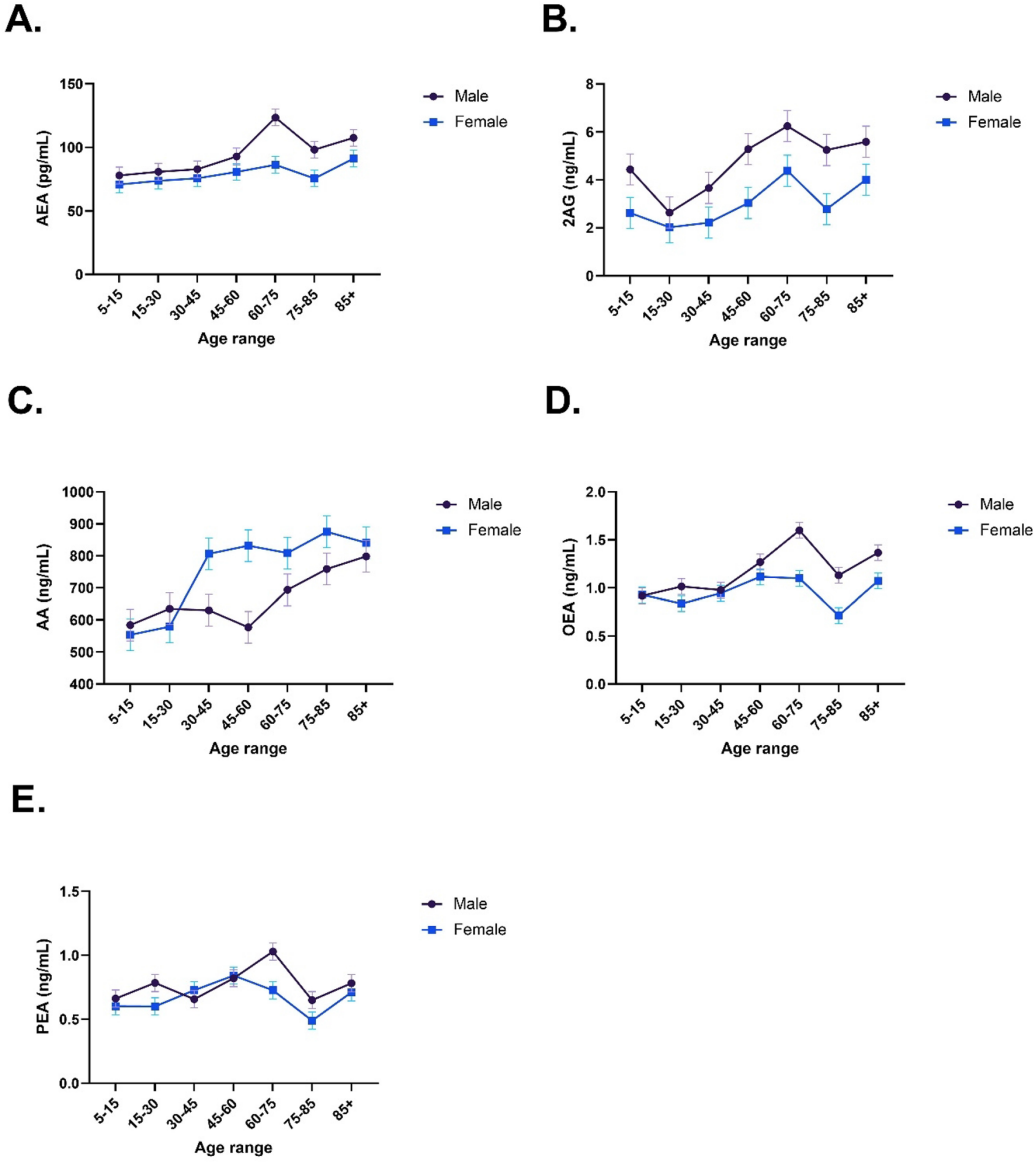
Average levels of serum arachidonoyl ethanolamide (AEA; **A**), 2-arachidonoyl glycerol (2AG; **B**), arachidonic acid (AA, **C**), oleoylethanolamide (OEA, **D**), and palmitoylethanolamide (PEA; **E**) levels across the lifespan, stratified by sex. Note: each data point is pooled serum from 50 participants. Error bars are standard error.

Separate univariate ANOVAs were conducted for each dependent variable. AEA levels increased significantly with older Age: *F*(6,14) = 6.12, *p* = 0.003, *η*_*p*_^*2*^ = 0.72 and significantly higher AEA levels were found in males (*M* = 94.80 pg/mL, *SD* = 17.51) compared to females (*M* = 79.14 pg/mL, *SD* = 9.26): *F*(1,14) = 19.97, *p* < 0.001, *η*_*p*_^*2*^ = 0.59, which seemed to occur largely after the age of 45 (Fig. 2A). However, the Sex × Age interaction did not reach significance: *F*(6,14) = 1.44, *p* = 0.269, *η*_*p*_^*2*^ = 0.38 (Fig. 2A).

2AG increased with older Age: *F*(6,14) = 5.08, *p* = 0.006, *η*_*p*_^*2*^ = 0.69 and was higher across the lifespan in males (*M* = 4.73 ng/mL, *SD* = 1.46) compared to females (*M* = 3.01 ng/mL, *SD* = 0.95): *F*(1,14) = 24.65, *p* < 0.001, *η*_*p*_^*2*^ = 0.64. No Sex × Age interaction was evident statistically: *F*(6,14) = 0.43, *p* = 0.847, *η*_*p*_^*2*^ = 0.16 or upon visual inspection of Fig. 2B.

AA (Fig. 2C) was significantly higher in females (*M* = 756.47 ng/mL, *SD* = 136.56) compared to males (*M* = 668.24 ng/mL, *SD* = 97.06): *F*(1,14) = 11.12, *p* = 0.005, *η*_*p*_^*2*^ = 0.44, which appeared to occur after the age of 30, though the Sex × Age interaction did not quite reach significance due to lack of power from the use of pooled samples: *F*(6,14) = 2.51, *p* = 0.073, *η*_*p*_^*2*^ = 0.52. As with the other ECBs, serum AA increased significantly with older age: *F*(6,14) = 7.57, *p* < 0.001, *η*_*p*_^*2*^ = 0.76.

OEA (Fig. 2D) also increased significantly with older Age: *F*(6,14) = 9.54, *p* < 0.001, *η*_*p*_^*2*^ = 0.80, and was higher in males (*M* = 1.18 ng/mL, *SD* = 0.25) compared to females (*M* = 0.96 ng/mL, *SD* = 0.17): *F*(1,14) = 26.04, *p* < 0.001, *η*_*p*_^*2*^ = 0.65, which only occurred after the age of 45 as evidenced by a near-significant Sex × Age interaction: *F*(6,14) = 2.67, *p* = 0.060, *η*_*p*_^*2*^ = 0.54.

Finally, PEA levels (Fig. 2E) were very similar to OEA, with higher levels in older Age: *F*(6,14) = 5.18, *p* = 0.005, *η*_*p*_^*2*^ = 0.69 and in males (*M* = 0.77 ng/mL, *SD* = 0.14) compared to females (*M* = 0.67 ng/mL, *SD* = 0.14): *F*(1,14) = 7.49, *p* = 0.016, *η*_*p*_^*2*^ = 0.35. There was no significant interaction between Sex and Age in the PEA data: *F*(6,14) = 1.80, *p* = 0.171, *η*_*p*_^*2*^ = 0.44.

### Correlations between measurements

Pearson’s coefficient correlations (*r*) were conducted between all measurements both across (Table 2) and within sexes (Tables 3, 4). As expected, ECB measurements tended to be highly positively correlated, except for between AA and other analytes. Interestingly, cortisol was significantly and positively correlated with AEA, OEA, and progesterone levels, but only in males. Conversely, progesterone was significantly and negatively correlated with 2AG, but only in females (Table 4).

**Table 2:** Pearson’s correlation coefficient values between ECBs and steroid hormones in pooled serum from males and females across the lifespan.

	AEA	2AG	AA	OEA	PEA	CORT	PROG
AEA	1	0.717***	0.237	0.879***	0.694***	0.614***	− 0.359
2AG		1	0.046	0.760***	0.504**	0.284	− 0.487**
AA			1	0.075	0.036	0.166	0.013
OEA				1	0.873***	0.478*	− 0.274
PEA					1	0.319	− 0.085
CORT						1	0.118
PROG							1

Sample size for each cell is *n* = 28 variable groups, which each consist of serum from 25 participants.

AEA = arachidonoyl ethanolamide, 2AG = 2-arachidonoyl glycerol, AA = arachidonic acid, OEA = oleoylethanolamide, PEA = palmitoylethanolamide, CORT = cortisol, PROG = progesterone.

*p < 0.05; **p < 0.01; ***p < 0.001.

**Table 3:** Inter-correlations between ECBs and steroid hormones in pooled serum from males across the lifespan.

	AEA	2AG	AA	OEA	PEA	CORT	PROG
AEA	1	0.570*	0.563*	0.914***	0.720**	0.786***	0.267
2AG		1	0.272	0.661*	0.38	0.401	0.071
AA			1	0.385	0.031	0.525	0.361
OEA				1	0.857***	0.657*	0.064
PEA					1	0.425	− 0.065
CORT						1	0.629*
PROG							1

Sample size for each cell is *n* = 14 pooled clusters, which each consist of serum from 25 participants.

AEA = arachidonoyl ethanolamide, 2AG = 2-arachidonoyl glycerol, AA = arachidonic acid, OEA = oleoylethanolamide, PEA = palmitoylethanolamide, CORT = cortisol, PROG = progesterone.

*p < 0.05; **p < 0.01; ***p < 0.001.

**Table 4:** Inter-correlations between ECBs and steroid hormones in pooled serum from females across the lifespan.

	AEA	2AG	AA	OEA	PEA	CORT	PROG
AEA	1	0.711**	0.603*	0.660*	0.559*	0.407	− 0.39
2AG		1	0.47	0.701**	0.443	0.087	− 0.582*
AA			1	0.258	0.303	− 0.07	− 0.216
OEA				1	0.890***	0.209	− 0.145
PEA					1	0.162	0.114
CORT						1	0.269
PROG							1

Sample size for each cell is* n* = 14 pooled clusters, which each consist of serum from 25 participants.

AEA = arachidonoyl ethanolamide, 2AG = 2-arachidonoyl glycerol, AA = arachidonic acid, OEA = oleoylethanolamide, PEA = palmitoylethanolamide, CORT = cortisol, PROG = progesterone.

*p < 0.05; **p < 0.01; ***p < 0.001.

### Principal component analysis

**Figure 3:**
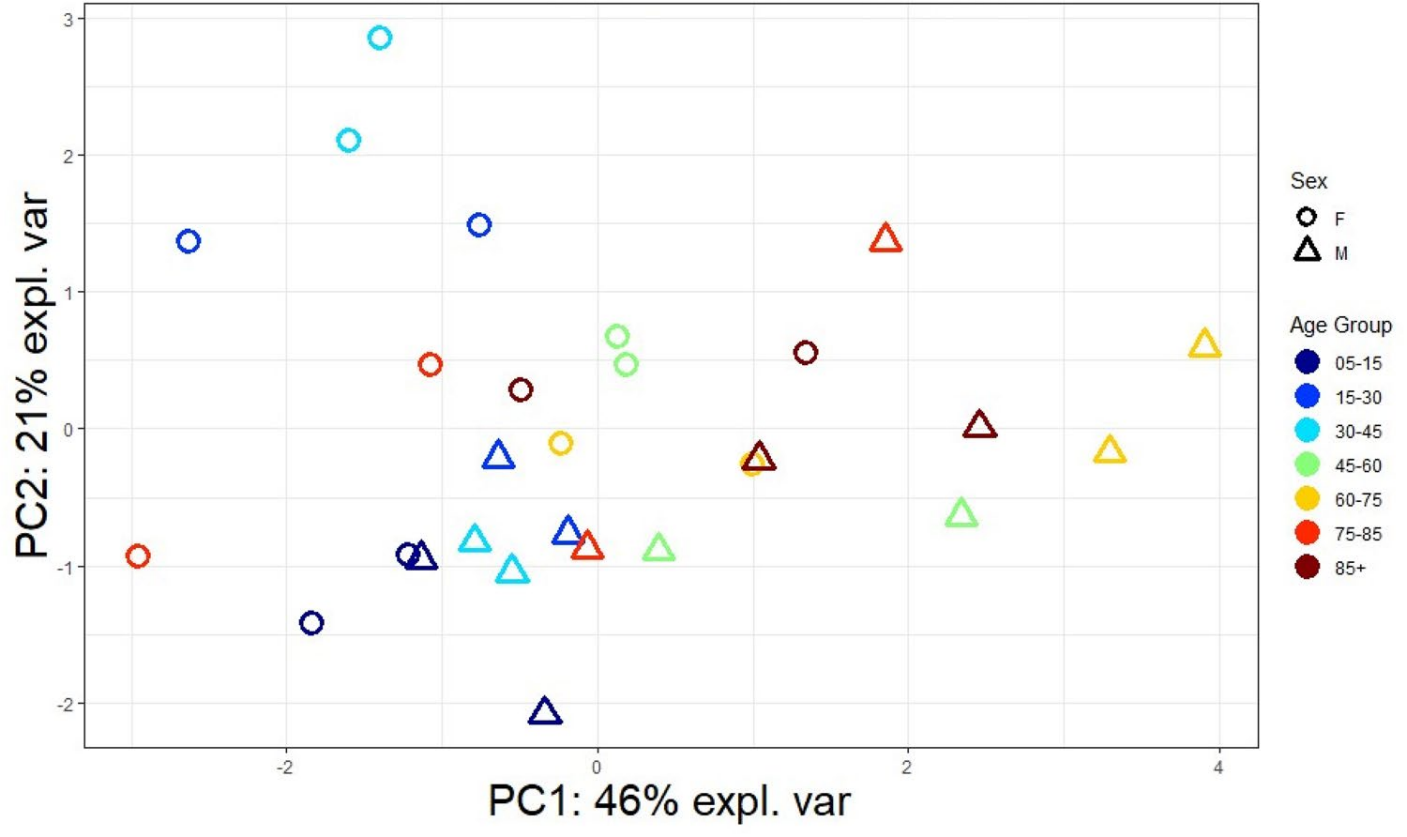
Unsupervised PCA plot on serum ECB sexual dimorphism across lifespan, grouped by age and sex. Note: each data point is pooled serum from a cluster of 25 participants. Data point colours and shapes corresponds to legend. M: Male; F: Female.

PCA plots were utilised for ECB and related hormones across the lifespan, to assess variability among the sexes and age groups (Fig. 3). When grouped by age, the two clusters of the same age and sex group were less likely to show variability, suggesting that there was less overall variability for each group. Interestingly, the clusters for both males and females in the 75–85 age group were uncommonly distant, suggesting that more variability between individuals occur in this age group. Females in the 30–45 age group and, to a lesser extent, in the 15–30 age group were notably distant from other groups and their male counterparts, as were males in the 60–75 age group. Therefore, females in their late teens and midlife years, and males in their older years, are more likely to show variability in their serum ECB and related hormone levels.

## Discussion

In the current study, we measured serum levels of ECBs, cortisol, and progesterone in 700 pooled samples stratified by age across the lifespan and sex. Confirming that the experiment was robust, serum progesterone showed the expected lifespan variability in females. Similarly, ECB and hormones across the age and sex groups visualised in the PCA plot showed expected variability. This is the first large-scale study to our knowledge of ECBs and related hormones across the lifespan to have reported age and sexual dimorphism, suggesting that these compounds correlate with known periods of major physiological changes in life. Therefore, the current study’s focus on ECBs and related hormones and their age and sex differences may have implications on neurological changes throughout the lifespan, and perhaps how these compounds could be further investigated to improve cognitive function and inform future pathological treatments.

The peak in female cortisol levels during the adolescent and early adult years may be due to pubertal development^33^. Additionally, the increase in male cortisol levels following the 30–45 age group occurred simultaneous to the general increases observed in the ECBs, with the exception of AA. Both cortisol and ECBs are influenced by the hypothalamic–pituitary–adrenal (HPA) axis^34^, with an increase in cortisol associated with increased ECB circulating levels^35^. The HPA axis alters and becomes less efficient with age^36^, so it is possible that the similar cortisol and ECB levels are confounded by HPA axis activation.

However, the changes in female cortisol levels are less straightforward, peaking in the 15–30 age group and slightly declining with age until it peaks again in the 85+ age group. Previous studies on the age-related trajectories of cortisol have reported conflicting results, with some showing increases over the lifespan^37,38^, and some showing decreases^39^. A recent study has even shown that cortisol levels over the lifespan follow a non-linear, U-shaped function^40^. Seeman et al.^41^ found similar cortisol responses reflected in the current study, with males aged 67–88 showing higher levels of cortisol than males aged 22–26. Though, the current study has utilised the broadest age range in available literature, with discernibility between males and females. As the secretion of cortisol and level of available cortisol activate the HPA axis, these results may have implications on the regulation of stress and neuroprotection in older age.

Oettel and Mukhopadhyay^42^ found that male progesterone levels remained stable and low throughout their lifetime, with no age-related changes reported. This was consistent with our data. However, in the current study, female progesterone levels greatly increased and peaked in the 30–45 age group, then decreased and plateaued starting from the 45–60 age group. The pubertal increase in female progesterone levels occurred simultaneous to the increase in female AA levels. Wilson et al.^43^ found evidence that progesterone modulates the release of AA, with the inhibition of progesterone increasing the release of AA. We observed that female AA levels increased greatly from the 15–30 age group to the 30–45 age group and continued to increase slowly but insignificantly from then onward. Female progesterone levels increased greatly until the 30–45 age group, and significantly decreased afterward. It is possible that our data reflects a direct relationship between female progesterone and AA, with loss of progesterone reducing the inhibition of AA release.

Serum progesterone in females showed the expected lifespan variability encountered with puberty onset (15–30), middle-end reproductive life and peri-menopause (30–45), and menopause (45–60)^44–46^. It may be possible that the use of hormonal contraceptives or hormone replacement therapy (HRT) impacted the detection of progesterone and cortisol, particularly in the younger females regarding the former. Furthermore, this may have altered HPA axis functioning. Combined hormonal contraceptives in particular inhibit the natural production of estrogen and progesterone, thereby eliminating menstrual-cycle-based variability^47^. This alters the HPA and the hypothalamic-pituitary-ovarian axis, preventing the maturation and release of an oocyte^48^ and inhibiting the rise in estrogen that usually occurs in the follicular phase^49^. Anovulation results in static progesterone synthesis in the ovary, which is then maintained at baseline levels^47^. Consequently, females that take hormonal contraceptives have lower serum progesterone than naturally cycling females^50^. Prior studies have also demonstrated that serum cortisol levels are typically elevated in people currently using oral contraceptives^51^.

Similarly, progesterone levels have decreased slightly in women taking menopausal progesterone-based HRT in comparison to menopausal women not taking HRT^52^. At least 29% of Australians choose to use an oral contraceptive pill^53^ and 38% of menopausal Australian women report having ever used HRT before^54^, which may suggest that these results were impacted by some samples that had elevated serum cortisol or lower serum progesterone levels due to the contraceptive pill or HRT.

There is more limited research on the effect of ECBs on the female reproductive system. Though, the fact that marijuana has severe consequences on pregnancy suggests that endogenous ligands of the receptors are involved in the modulation of pregnancy, menopause, and the menstrual cycle^55^. AEA has been quantified in human follicular fluid^56^, in which its levels strongly correlated to oocyte maturation and quality in women undergoing infertility treatment^57^. When quantifying levels of AEA across pregnancy, it was also found that AEA levels were high in the first trimester – similar to levels measured in the luteal menstrual phase—and increase almost two- to four-times more with the onset of labour^58,59^. Therefore, ECB levels are most likely to fluctuate during a time of pregnancy, which may partially explain the slight increase of ECB levels in females in the 15–30 age group. The highest number of pregnancies occur in women’s twenties, with an increasing number of pregnancies occurring in women in their thirties^60^. Due to the unidentifiable nature of the pathology test data collection, the current study is unable to discern pregnant females within the study population. Though, these results may have implications on female reproduction and fertility, and how ECB levels may affect these. Future research should monitor ECB levels over the course of pregnancy to further investigate this.

Additionally, female menopause and post-menopause are partially affected by ECB levels. The activity of menopause, and the end of the menstrual cycle fluctuations, were significantly associated with increased 2-AG levels in a metabolic parametric study^61^. Postmenopausal AEA levels were also found to be similar to those in the first trimester of pregnancy and the luteal menstrual phase, which were significantly higher than AEA levels in the later trimesters^58^. Similarly, the current study found that females in the 40–60 age range had higher ECB levels compared to younger age groups. Additionally, females in the 60–75 age group, where post-menopause is most experienced, recorded a peak increase in AEA and 2-AG levels. Therefore, as the activities of the menstrual cycle cease in mid-life, ECB levels tend to increase to a peak. This may have potential implications on how ECBs are neuroprotective in later life^62^ and, more broadly, have a role in neurological decline.

AA levels trended differently to the other ECBs; as age increased, AA levels were higher in females compared to males. In contrast, as age increased, AEA, 2-AG, OEA, and PEA were higher in males compared to females. Similar results were found by Lohner et al.,^20^ in which AA was significantly lower in males compared to females, across the age groups. Additionally, AA was significantly higher in females aged 51 years and older, with an insignificant increase in females aged 13 to 50 years old, compared to males. This may be because AA, in comparison to the other ECBs, is also considered a long-chain polyunsaturated fatty acid. Recently, there has been growing evidence of a complex interplay between the ECB system and the long-chain polyunsaturated fatty acids^62^. Notably, both AEA and 2-AG are derived from AA^63^. If there is a high amount of AA available in one’s body, it can potentially be converted into comparatively more AEA and 2-AG. Similarly, when these ECBs are no longer needed, they are rapidly degraded back into AA^62^. Therefore, it may be suggested that if AA is high, AEA and 2-AG may be lower. This can be further evidenced by findings of inversely proportioned 2-AG and AA in mice^64^. For the current study, this may explain that while AA was higher in females than males, AEA and 2-AG were observed to be comparatively lower. Additionally, female progesterone levels were negatively correlated with 2-AG levels. As female progesterone levels spiked during puberty and pre-menopause, 2-AG levels remained low. In older years, progesterone decreased while 2-AG levels increased. This may indicate progesterone regulation of 2-AG (or vice versa), or may simply be a coincidence given that 2-AG also increased in older age in men. Potentially, the rate of metabolism of AA into its metabolites 2-AG and AEA may change over the lifespan, and it is possible that this is influenced by changes in sex hormones with increased age. Further research into this question may have implications in neurogenesis and brain development across the lifespan^65^.

A study by Fanelli et al.^66^ may suggest an alternative confounding explanation of our ECB results. By measuring body mass index (BMI) and the metabolic parameters of normal, overweight, and obese adults, Fanelli et al.^66^ suggested that N-acylethanolamines play specialised roles in the body’s energy balance. AEA influenced energy intake and storage and became a biomarker for abdominal fat, while OEA mediated anorectic signals and lipid oxidation. Additionally, increased AEA in men and low OEA in men and menopausal women were also associated with low HDL-cholesterol. Abdominal fat and low HDL are highly characteristic of obesity^67^. The likelihood of obesity peaks between 40 to 75 years old^68^ and is more prevalent in older males than older females^69^. Additionally, ECBs increase and become overactive in obese individuals^70^. This was reflected in our results, as AEA, 2-AG, and OEA peaked in older males 60–75 years old. These ECBs also remained higher in males across almost all age ranges, compared to females. Therefore, obesity was a potential confounding factor that influenced the age and sex-related effects on ECBs.

Male testosterone levels across the lifespan potentially relates to the ECB levels recorded in the current study. Previous animal studies have provided direct evidence of the ECB system in the testis, thereby implicating sex-related differences and an effect on testosterone production^71–73^. In marijuana users with a highly expressed ECB system, it was found that plasma testosterone was reduced in comparison to non-marijuana users^74^. Therefore, when ECB levels are elevated, testosterone levels are decreased. Additionally, AEA was found to downregulate luteinising hormone in the male anterior pituitary^75,76^. Luteinising hormone upregulates testosterone production^77^, which suggests that AEA activity leads to a decrease in testosterone production. Testosterone levels are found to peak in late teen years, and thereby steadily and slowly decrease over the lifespan, and more readily decrease after 80 years old^78–80^. In the current study, male ECB levels trend upwards, with most ECBs peaking in the 60–75 age group and dropping in the 75–85 age group before slowly increasing again. Interestingly, AEA and OEA levels were also found to be significantly positively associated with cortisol, only in male serum. The addition of testosterone as a measurable variable may have allowed better understanding of the relationship between male cortisol levels and ECB levels. It is possible that as male testosterone levels slowly decrease across the lifespan, ECB levels slowly increase in association. Future research should look at broader sex-related hormones and ECBs across the lifespan to specifically investigate male sex-related changes, and a potential implication on male sex-related diseases such as prostate cancer and testicular cancer.

To date, the current study is the largest on this topic, with a broad range of under-researched ECBs and related hormones. The results from this study could have implications on how ECBs affect age-related diseases and declines, especially as ECBs are mostly produced in the brain. Therefore, the progression of ECB levels across the lifespan could inform understanding of neurological diseases, such as dementia, brain cancer, and Parkinson’s disease. ECBs are also produced in various muscles, fatty tissues, and immune cells, and thus the current results have implications on broader affected pathways. As ECBs are related to sex hormones, further research should be conducted on how the expression of ECBs and a more exhaustive list of sex hormones develop over the lifespan. Specifically, further investigating testosterone could inform further understanding of the changes in male ECB levels.

As one of the first investigations of this size, it is important for future studies to expand on the results of the current study. Pooling the data in the current study enabled a large sample size of highly generalisable data from the general population, but due to the deidentifying nature of pathology test data collection, no demographics or possibly confounding health conditions were recorded. Therefore, it cannot be truly concluded that these ECB, cortisol, and progesterone levels reflect actual changes across one’s lifetime. Additionally, 2-AG and PEA levels were significantly positively associated in the pooled analysis of male and female sera, but no longer reached significance once stratified by sex. It may be possible that separate lifespan changes for males and females may have affected these results, such as neurological decline, female menopause, or obesity, though the experimental design of the current study has limited the ability to discuss these implications. Future studies should be encouraged to build upon these results to further understand the sex- and age-related affects on 2-AG and PEA, amongst related ECBs. Ideally, the sample should be followed throughout their lifetime with their analyte levels recorded consistently. Though, the sampling of the current study utilises one of the most readily available and convenient methods. Additionally, due to the wide scope and unanimity of pathology sampling in Australia, a considerable portion of blood tests are taken by healthy individuals for other routine non-pathological reasons, such as immunology, nutrition, pregnancy, or medication. Any effect that undisclosed health conditions may have had on the results were most likely insignificant.

It is also noted that, due to sample pooling, diurnal variations of cortisol and cyclic variation of progesterone in females could not be analysed. There is evidence of changes of ECB, progesterone, and cortisol levels across age strata mirroring lifespan milestones such as puberty and female menopause, though not across different times of the day or ovarian cycle. Peak cortisol levels are recorded in the morning and decrease to its lowest level late at night^81^, while progesterone is decreased during the follicular phase and increased in the luteal phase^82^. Though, research on cortisol diurnal intensity and progesterone cyclic intensity across the lifespan is limited. To compare the activity and adaptations across the lifespan, it is recommended that these patterns are further investigated across age and sex in the future.

The current study finds evidence for changes in ECB, cortisol, and progesterone serum levels across age that were substantially affected by participant sex. These findings add to our knowledge of the relationship between ECBs and sex hormones as well as life changes, and support the theory that ECBs show sex-dimorphism across the lifespan.

## Data Availability

The data that support the findings of this study are available from Sullivan Nicolaides Pathology, Australia, but restrictions apply to the availability of these data, which were used under license for the current study, and so are not publicly available. Data are however available from the authors (please contact luke.ney@qut.edu.au) upon reasonable request and with permission of Sullivan Nicolaides Pathology, Australia. Otherwise, most data generated and analysed during this study are included in this published article.
